# ATG Exposure in T Replete Transplant for Hematological Malignancies: Real World Analysis from BMT CTN 1202

**DOI:** 10.21203/rs.3.rs-9918950/v1

**Published:** 2026-06-17

**Authors:** Madhavi Lakkaraja, Audrey Mauguen, Kinga Hosszu, Devin McAvoy, Valerie Greco-Stewart, Gabrielle Schmidt, John Levine, Miguel Angel Perales, Sergio A. Giralt, K Scott Baker, Jaap Jan Boelens

**Affiliations:** Fred Hutch Cancer Center; Memorial Sloan Kettering Cancer Center; Memorial Sloan Kettering Cancer Center; Memorial Sloan Kettering Cancer Center; NMDP; CIBMTR (Center for International Blood & Marrow Transplant Research), NMDP; Icahn School of Medicine at Mount Sinai; Memorial Sloan Kettering Cancer Center; Memorial Sloan Kettering Hospital Cancer Center 1275 York Avenue; Fred Hutchinson Cancer Research Center; Memorial Sloan Kettering Cancer Center

**Keywords:** Hematopoietic stem cell transplant, T-replete, rabbit anti-thymocyte globulin, Model-based-dosing, BMT CTN 1202

## Abstract

Traditional weight-based dosing results in variable rabbit anti-thymocyte globulin (rATG) clearance, delaying CD4 + Tcell-immune-reconstitution (CD4 + IR) and impacting outcomes. In a retrospective pharmacokinetic/pharmacodynamic analysis of patients undergoing first T-replete hematopoietic cell transplantation (HCT), enrolled on BMT CTN 1202, we estimated post-HCT rATG-exposures as area under the curve (arbitrary unit per day/milliliter [AUxd/mL]) using a validated pharmacokinetic-model. Results were compared to patients who did not receive rATG. Previously defined post-HCT rATG-exposure groups: A:<30, B:30–55 and C:≥55 AUxd/mL were correlated with outcomes of interest using cox-proportional-hazard and cause-specific-hazards models.

325 patients (median age 51 years) were included: 228 received rATG. Median post-HCT rATG-exposure was 44.1 (Range 6.2–125.0). Among patients who received rATG, higher exposure correlated with worse five-year overall survival (OS) (no-ATG:51%; group A:67%; B:49%; C:34%, p = 0.01), higher five-year relapse incidence (no-ATG:31%; A:27%; B: 42%; C: 58%, p < 0.001) and lower CD4 + IR (no-ATG:64%; A:73%; B:51%; C:19%, p = 0.001). Grade 2–4 acute graft versus host disease (GVHD) rates were: no-ATG:40%; A:26%, B:39%, C:35%, (p = 0.44). Any rATG-exposure was associated with lower moderate/severe chronic GVHD (HR:0.63, p = 0.025).

Low post-HCT rATG-exposure (< 30AUxd/mL) but not absent correlated with higher OS, lower relapse, and lower GVHD related deaths. Model-based-dosing could be used to target optimal post-HCT rATG-exposure and improve HCT outcomes.

## Introduction

Allogeneic hematopoietic cell transplantation (HCT) is an effective and potentially curative treatment modality for hematological malignancies. With advancement of supportive care, human leukocyte antigen matching, and graft versus host disease (GVHD) prevention, the outcomes after HCT have improved remarkably. Despite significant strides made over the last few decades, transplant related mortality (TRM), and relapse of disease remain major causes of morbidity and mortality following HCT.^1^

Early CD4 + immune reconstitution (CD4 + IR) reduces risk of infection, TRM, chronic-GVHD (cGVHD) and subsequently increases overall survival (OS).^2–5^ Rabbit antithymocyte globulin (rATG) is used in conditioning regimens to reduce risk of graft rejection and GVHD.^6^ Traditional weight based dosing (mg/kg) of rATG results in highly variable exposures of rATG post-HCT. Admiraal et al., developed and validated a population pharmacokinetic (PK) model for rATG which demonstrated that weight [< 40 kilograms (kg)] and absolute lymphocyte count (ALC) are main predictors for rATG clearance.^7,8,9^ Post-HCT exposures of rATG predicts outcomes; with higher rATG exposures leading to delayed CD4 + IR and inferior outcomes.^10^ Studies, in Europe and United States (US), in different HCT platforms (bone marrow, cord, T-cell-depleted setting, and T-replete setting), demonstrated that “optimum” exposure of rATG is crucial for favorable HCT outcomes. ^7–12^

Prospective clinical trials in Europe (PARACHUTE) and US (PRAISE IR) demonstrated that PK ‘model-based dosing’ (MBD) of rATG resulted in higher attainment of early CD4 + IR, higher OS and lower TRM in T-replete pediatric HCT (bone marrow and cord blood) and ex-Vivo (CD34+-selection) T-deplete setting respectively.^13,14^ Recent studies showed that MBD of rATG had a beneficial impact on TRM, OS, viral reactivations, graft-failure and cGVHD.^15–17^ However, these analyses mainly included pediatric and young adult patients. There is little data on the impact of rATG exposure on outcomes in adult patients with hematological malignancies undergoing T-replete HCT and comparisons to patients without rATG-exposure has yet to be performed.

In this study, we assessed the impact of exposure to rATG (including none) in a cohort of patients with hematological malignancies from a central database and repository, the Blood and Marrow Transplant Clinical Trials Network (BMT CTN) 1202 study to test hypothesis that an “optimum” rATG exposure improves T-replete HCT outcomes in patients with hematological malignancies.

## Materials/Subjects and Methods

A retrospective pharmacokinetic and pharmacodynamic (PK-PD) analysis of data from patients enrolled on BMT CTN 1202 with hematological malignancies who underwent their first T-replete HCT and received rATG (thymoglobulin) as part of conditioning was performed. The dosing and schedule of rATG was based on institutional practice, details of which are not available. Patients fulfilling the same eligibility criteria but did not receive rATG who were enrolled in the BMT CTN 1202 study were included as a ‘no-ATG’ group in the analysis (**Supplemental Fig. 1**). The BMT CTN 1202 trial - “Prospective Multi-Center Cohort for the Evaluation of Biomarkers Predicting Risk of Complications and Mortality Following Allogeneic HCT” was established to longitudinally collect comprehensive, standardized, clinical data.^18^

Data from Center for International Blood and Marrow Transplant Research (CIBMTR) database and BMT CTN 1202 dataset were used to simulate post-HCT rATG exposures (after day 0) as area under the curve (AUC) (arbitrary unit per day/milliliter [AUxd/mL]) by using a validated population-PK model (Admiraal et al, 2017), accessed through the InsightRx platform (San Francisco, California).^9^ The following parameters were inputted for each patient in the model to estimate rATG-exposure: date of HCT, weight, height, date of first rATG dose, dose of rATG, length of rATG infusion, number of days of infusion, and ALC rATG dosing. In patients where ALC was not available on the day of administration of rATG, the following was considered: (a) Patients who received rATG on days − 4,−3,−2, or −1, ALC was considered as 0.1 .(b) pPatients who received rATG earlier than day-7, actual reported values were used. (c) Patients who received rATG on day-5 and day-6, ALC was reported within three days of start of rATG were used.

The BMT CTN 1202 study was approved by the instiutonal review board (IRB) at all participating centers. This study was approved by the IRB at Fred Hutch Cancer Center (FHCC) and Memorial Sloan Kettering Cancer Center (MSKCC), (FHCC IRB:6007 − 2186 and MSKCC IRB:X22–032) and conducted in accordance with the Declaration of Helsinki.

### Outcomes:

The main outcome of interest was OS defined as the time from HCT to death from any cause. Surviving patients were censored at their date of last contact. CD4 + IR was defined CD4^+^>50/uL at Day + 100 and analyzed as a binary endpoint. For CD4 + IR, CD4 + counts (when available) as reported in the database were used; these were typically reported at 100 days, 6 months and 1 year post-HCT. In this study, “early CD4 + IR” wasdefined as CD4 + > 50/uL at day + 100. Patients alive with missing CD4 + value at 100 days or patients lost to follow-up before 100 days (n = 207) were excluded from the analysis while patients who died within 100 days (n = 35) were included in analysis as lacking early CD4 + IR. Other outcomes of interest were relapse, acute-GVHD (aGVHD), chronic GVHD (cGVHD, moderate/severe), and TRM. Time-to-relapse was defined as time from HCT to disease relapse. Patients without relapses were censored at their date of last follow-up while deaths before relapse were treated as competing events. Times to aGVHD or cGVHD are defined as the time from HCT to the apparition of the GVHD. Patients without GVHD were censored at their last follow-up while death or relapse before GVHD were treated as competing events.

Patients alive are censored at their last follow-up. TRM was defined as the time from transplant to death related to treatment. Patients alive are censored at their last contact, while relapse and death not related to treatment were counted as competing events. Primary graft failure was defined as failure to achieve neutrophil engraftment by day 30 after HCT. Causes of death were captured as per CIBMTR guidelines.

### Statistical analyses:

Post-HCT rATG exposures, using previously defined thresholds (Group A:<30, Group B:30–55, Group C:≥55 AUxd/mL),^19^ were correlated with outcomes of interest: OS, CD4 + IR, defined as CD4 + > 50/uL by Day + 100, TRM, aGVHD2–4, cGVHD, and relapse using CIBMTR definitions.

Cox proportional hazard and cause-specific hazards models adjusted on clinical factors were used to assess previously defined AUC groups while splines and maximally selected log-rank statistics were used to search for new thresholds. For time-to-event endpoints OS, survival rates were estimated using Kaplan-Meier estimator, while for endpoints in presence of competing risks (aGVHD, cGVHD, relapse and TRM), the cumulative incidences were estimated using Aalen-Johansen estimator. The impact of rATG exposure (using pre-defined groups; No-ATG, < 30, 30–55, > 55), and other factors (age at HCT, sex, graft source, receipt of TBI-based conditioning regimen, myeloablative vs. reduced intensity/non-myeloablative conditioning) on outcomes were estimated using Cox proportional models and cause-specific hazard models when in presence of competing risks. The impact of reaching CD4 + IR on the risk of TRM was assessed using a landmark analysis from Day + 100 post-HCT. The results are presented as hazards ratios (HR), 95% confidence intervals (95%CI) and log- likelihood test p-values. The impact of rATG exposure on CD4 + IR was assessed using logistic regression, and odds ratios (OR) with 95%CI.

In an exploratory analysis using splines and maximal log-rank statistics we checked if previously defined thresholds were applicable to the current data as well.

#### Data Sharing Statement

Data transfer agreements were done between CIBMTR/NMDP MSKCC and FHCC and a deidentified dataset was provided.

## Results

### Patient Characteristics

**Figure 1:**
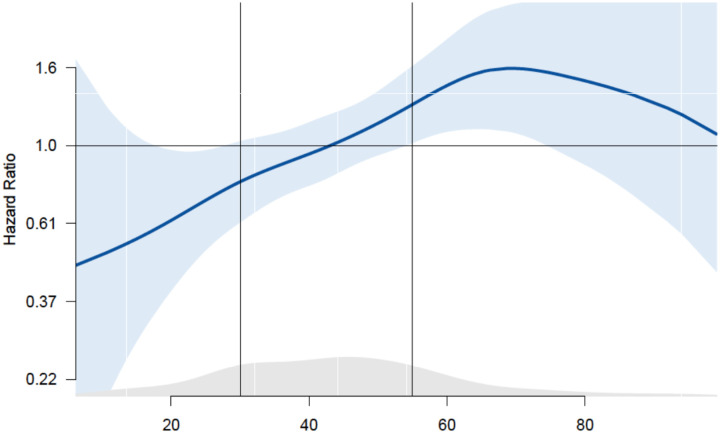
Optimum rATG exposure thresholds. HR for rATG exposure by OS. Optimum rATG exposure thresholds of ~30 AUxd/mL can predict outcomes. OS: Overall survival, rATG: rabbit anti-thymocyte globulin, HR: Hazard Ratio, HCT: Hematopoietic Stem Cell Transplantation.

We identified 325 patients who underwent their first T-replete allo-HCT for a hematological malignancy on BMT CTN 1202 study. Median age was 51 (range 4 to 74) years, 303 (93%) patients were > 18 years old, and 139 (43%) patients were female. In this cohort, 196 (60%) patients received myeloablative conditioning. Graft source was peripheral blood in 239 (74%) patients, and bone marrow in 86 (26%) patients. Of these 325 patients, 228 received rATG and 97 did not. Median rATG dosing was 5 mg/kg in patients who received rATG and median start day of rATG from HCT was − 3 days (range: −11 to −1). In patients who received rATG, median post-HCT exposure was 44.1 (range 6.2 to 125.0) AUxd/mL, and median ALC count was 0.1 (range 0.1 to 3.3). Detailed patient and HCT characteristics are presented in Table 1. We confirmed the optimum threshold of post-HCT rATG exposure influencing HCT outcomes was around 30 AUxd/mL, as noted in our prior studies (Fig. 1).

### Outcomes of interest

**Figure 2:**
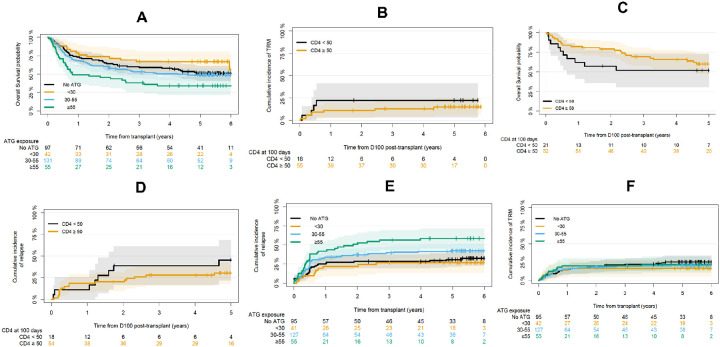
Outcomes by rATG exposure. **A,** OS and rATG exposure. **B,** TRM and Early CD4+IR. **C,** OS and Early CD4+IR. **D,** Relapse and Early CD4+IR. **E,** Relapse and rATG exposure. **F**, TRM and rATG exposure. Post-HCT rATG exposure <30 AUxd/mL was associated with higher OS, and lower relapse. OS: Overall survival, rATG: rabbit anti-thymocyte globulin, TRM: Transplant Related Mortality, CD4+IR: CD4+ immune reconstitution defined at CD4+>50 by Day +100 post transplant

The five-year OS declined with increasing post-HCT rATG: Groups A: 67%; B: 49%; C: 34%, while for the ‘no-ATG’ group OS was 51%, p = 0.007; (Table 2a, Fig. 2a). The other predictor for OS was age at HCT (adjusted HR per year: 1.02, 95%CI: 1.01, 1.03, p < 0.001, Table 2a). A higher rATG exposure correlated with a lower rate of CD4 + IR (Groups A: 73%; B: 51%; C: 19%, while for no rATG group CD4 + IR was 64%, p = 0.003; (Table 2b). The other predictor for CD4 + IR was graft source, with patients receiving PBSCs more likely to attain CD4 + IR at day 100 post-HCT compared to bone marrow (56% vs. 41%; adjusted OR: 3.24, 95%CI 1.16, 9.97, p = 0.02). Patients who attained early CD4 + IR had TRM of 15% and patients who did not attain early CD4 + IR had TRM of 22% (Fig. 2b). Patients who attained early CD4 + IR had OS of 60% and patients who did not attain early CD4 + IR had OS of 52% (Fig. 2c). Patients who attained early CD4 + IR had relapse rate of 30% and patients who did not attain early CD4 + IR had relapse rate of 45% (Fig. 2d).

Higher rATG exposure was associated with increasing 5-year relapse rate (Groups A: 27%; B: 42%; C: 58%, while for no-ATG group relapse probability was 31%, adjusted HR for C vs No-ATG: 2.73, 95%CI: 1.58, 4.73, p < 0.001; Table 2c, Fig. 2e). Hazard of relapse was also increased with higher age at HCT (adjusted HR per year: 1.02, 95%CI: 1.00, 1.13, p = 0.007) and with TBI use in conditioning (adjusted HR for TBI vs no TBI: 2.18, 1.21, 3.91, p = 0.01; Table 2c). There was no significant difference in cumulative incidence of grade 2–4 aGVHD and grade 3–4 aGVHD between the four exposure groups (**Supplemental Table 1**). The incidence of extensive cGVHD, was lower in patients with any rATG exposure compared to no-rATG (HR: 0.63, 95%CI: 0.42, 0.94, p = 0.025; **Supplemental Fig. 2a, 2b, 2c, 2d**).

Although not statistically significant, in patients that received rATG, increasing rATG was associated with higher TRM (Groups A; 17%, B; 21%, C; 22%), while no-rATG group had highest TRM (25%) (p = 0.48; Table 2d, Fig. 2f). Higher age at HCT was associated with higher hazard of TRM (HR: 1.03, 95%CI: 1.01, 1.05, p = 0.002; Table 2d).

**Figure 3:**
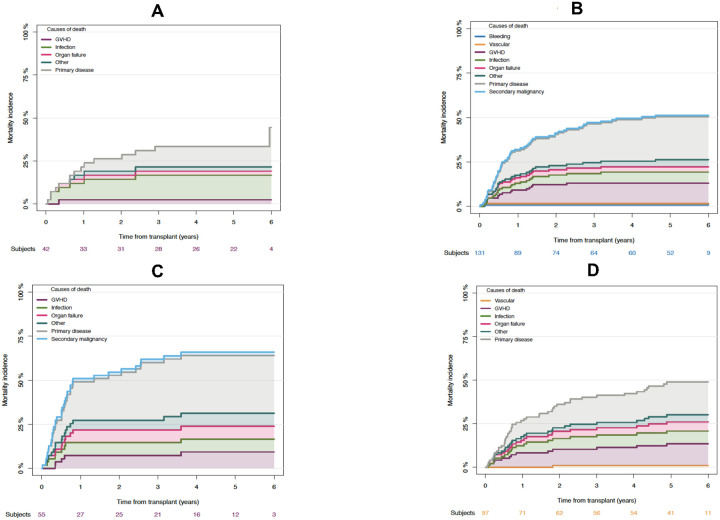
Cause of Death by Post-HCT rATG exposure **A,**Post-HCT rATG <30 AUxd/mL. **B,** Post-HCT rATG 30–55 AUxd/mL. **C,**Post-HCT >55 AUxd/mL. **D,** No ATG. rATG: rabbit anti-thymocyte globulin, HCT: Hematopoietic Cell Transplantation, GVHD: Graft versus Host Disease

The causes of death for 164 patients who died was primary disease in 73 (45%), GVHD in 33 (20%), infection in 25 (15%), toxicity or organ failure in 14 (9%), other causes in 14 (9%), secondary malignancy in 2 (1%), vascular causes in 2 (1%) and bleeding in 1 (1%) patients (Table 3, Fig. 3). GVHD related mortality was lowest in the < 30 group; 2%, compared to 12% in group B (30–55), 9% in group C (≥ 55) and 13% in no-rATG group. In patients who attained early CD4 + IR, 2 deaths were noted secondary to ‘other’ causes. The rates of neutrophil engraftment was similar in all groups (98% in Groups A, B, C and 99% in No-ATG group).

## Discussion

In this large ‘real world’ analyses of patients who participated in a prospective registry trial (BMT CTN 1202), we simulated rATG exposures post-HCT using a validated population-PK model (Admiraal et al).^9^ A previously described post-HCT rATG exposure of < 30 AUxday/mL correlated with higher OS, faster CD4 + IR and lower relapse probability, compared to higher post-HCT rATG exposures (30–55 and ≥ 55 AUxd/mL) and patients who did not receive ATG.^7,9,14,19,20^ The difference in OS between low rATG exposure and no-ATG was mainly driven by higher GVHD related deaths in the ‘no-ATG’ group. Most importantly, this real world study is the first to assess the rATG exposures in context with a “No-ATG” group, and assess the impact of rATG exposures on outcomes in a cohort of mainly adult patients with hematological malignancies who underwent HCT.

Prior single center studies that assessed effect or rATG exposure on HCT outcomes demonstrated that lower post-HCT rATG exposure leads to improved CD4 + IR at day + 100, associated with lower TRM and subsequently higher OS.^4,9,19,20^ Early CD4 + IR (before day + 100) can also impact cGVHD, and was demonstrated in a tri-institutional analyses.^21^ This effect was also seen in a prospective single-arm phase-2 trial (PARACHUTE trial) in the pediatric population, and in a recent multi-center update.^14,15^ In these studies, MBD of rATG was compared to a historical cohort of patients who received traditional weight based dosing of rATG. Patients who received MBD of rATG attained earlier CD4 + IR (defined as CD4 + > 50/uLx2 within the first 100 days) and had higher OS compared to the historical cohort.^14^ In the updated analysis (including patients enrolled on the trial combined with real world data of two centers) of 214 patients, the MBD group had a 3-fold lower incidence of cGVHD, graft failure, and viral reactivations, and the difference in OS was driven by CD4 + IR, and not by transplantation era.^15^

Another study using the same MBD nomogram as in PARACHUTE of rATG showed similar favourable HCT outcomes in pediatric patients with malignant and non-malignant disorders.^16^ In a mainly adult (> 80%) CD34 + T-cell-depleted setting, it has been demonstrated that rATG exposure affects outcomes, including CD4 + > 50/ul at day 100, NRM, and survival. In a subsequent phase 2 prospective trial (PRAISE IR), using MBD dosing targeting to a low post-HCT rATG exposure (< 20 AUxd/mL) led to faster CD4 + IR, lower TRM and higher OS.^17,19^ The MBD nomogram used here was the same as in PARACHUTE, but rATG was started on day − 12 instead of day − 9. In line with these results, in children undergoing T-cell-depleted HCT rATG exposure < 20 AUxd/mL was associated with better clinical outcomes.^22^ Furthermore, in children and young adults with hematological malignancies who underwent αβ-T-cell-depleted haploidentical HCT, similar outcomes and same optimal post-HCT exposure was noted.^23^

The current study is unique in that it not only validates the PK-PD-model, but it also adds to the literature as it described the findings in mainly an adult T-replete cohort with hematological malignancies. The study compared the 3 previously described rATG exposure groups to a ’no-ATG’ group of 97 patients who did not receive rATG, which has not been done before. This ‘no-ATG group’ was from the same registry - BMT CTN 1202 study. Thus we found that lower rATG exposure after HCT is associated with better HCT outcomes, but omitting rATG results in worse outcomes. This suggests that other mechanisms than T-cell-depletion may have a role. Previously it was found that a higher pre-HCT rATG exposure is associated with lower rates of GVHD.^20^ Given the polyclonality of rATG (including targets to dendritic cells) a potential mechanism may be dendritic cell depletion which leads to decrease in antigen presentation, which plays a key role in initiating GVHD. Alternatively, rATG may ‘cool off’ inflammation in target organs for GVHD, subsequently reducing antigen presentation.

In this current study, we observed that higher rATG post-HCT exposure was associated with increased rate of relapse. This impact of rATG exposure on relapse, was not seen in prior studies, which had a significant pediatric population, where the main impact of post-HCT rATG exposure was observed on TRM. A possible explanation for this may be that in pediatric population, patients are heavily pretreated and thus are mostly minimal residual disease (MRD) negative prior to proceeding to transplant. Adult patients may need to rely more on ‘graft-versus-leukemia’ effect from transplant, which is likely compromised with higher post-HCT exposure of rATG. We were unable to analyse the impact of MRD or pre-transplant chemotherapy, in this cohort, due to limited data.

In this study we considered “early CD4 + IR”, defined as CD4 + counts > 50 at day + 100. CD4 + counts > 50 at least twice by day + 100 was used as definition of early CD4 + IR in most studies, and was repeatedly found as predictor for outcomes.^3,16,19–21^ In a recent study using MBD of rATG CD4 + > 50 by day 90 was used as a surrogate marker for early CD4 + IR. ^16^

The primary limitations of this study is that it is retrospective in nature. In addition, we had limited CD4 + IR data which made detailed analyses on impact outcomes challenging. Also, ALC data was limited in some patients, and counts were taken as expected based on day of rATG administration consistent with literature and clinical findings as described in methods section. We saw a trend where rATG exposure < 30 AUxd/mL seemed to be associated with lower TRM, consistent with other studies, although the finding was not statistically significant due to lack of power. Differences in this mainly adult cohort were mainly driven by higher relapse probability in the higher rATG exposure groups. This study was a registry based study where data from different insititutions was pooled, and the investigators only had access to deidentified data, thus validating prior single and combined center studies.

Precision MBD dosing of rATG targeting to an optimal exposure post-HCT may help in better predicting CD4 + IR and subsequently increasing survival chances. Findings of this registry-based analysis confirms that targeting rATG to lower exposure but not absent exposure, post-HCT may improve outcomes of patients with hematological malignancies undergoing T-replete HCT. MBD of rATG is an easy and cost effective method which can be implemented widely in different settings globally including in low / middle income countries (as the study from Barriga et al suggests).^16^ The dosing nomogram (used in various trials:PARACHUTE and PRAISE IR) has been extrapolated from the validated population-PK model recommends dose of rATG and start-date of rATG based upon weight and ALC, stem cell source and type of HCT. It is an easy tool to use and can be used even by centers who do not have access to the Insight Rx platform. An upcoming trial, the BMT CTN 2502 “IMPACT” study will assess MBD of different conditioning agents including this nomogram for rATG in children and young adults with inborn errors of immunity undergoing HCT. In conclusion, MBD dosing of rATG can be used to improve HCT outcomes, laying the foundation for wider applicability.

## Supplementary Material

Supplementary Files

This is a list of supplementary files associated with this preprint. Click to download.

- SupplementalTable1.pdf
- SupplementalFigure1.pdf
- SupplementalFigure2.pdf
- Tables.docx

## Key Points

1. Low post-HCT rATG-exposure (< 30 AUxd/mL) but not absent is associated with higher OS, lower relapse, and lower GVHD related deaths.
2. Model based dosing could be used to target optimal post-HCT rATG exposure and improve HCT outcomes.
